# Bilateral osteochondrosis of the distal tibial epiphysis: a case report and review of the literature

**DOI:** 10.3389/fped.2025.1586953

**Published:** 2025-09-25

**Authors:** Victor Aye, Maxime Pilloux, Elio Paris, Dimitri Fasel, Giacomo De Marco, Oscar Vazquez, Christina Steiger, Romain Dayer, Sana Boudabbous, Dimitri Ceroni

**Affiliations:** ^1^ Faculty of Medicine, University of Geneva, Geneva, Switzerland; ^2^ Pediatric Orthopedics Unit, Pediatric Surgery Service, Geneva University Hospitals, Geneva, Switzerland; ^3^ Radiology Department, Geneva University Hospitals, Geneva, Switzerland

**Keywords:** paediatric, osteochondrosis, distal tibia epiphysis, bilateral, sportive activity

## Abstract

Osteochondrosis of the distal tibial epiphysis is a rare condition, and its clinical presentation thus remains poorly defined. The few reported cases in the literature suggest an insidious, progressive onset of ankle pain and swelling with no clear history of trauma. Imaging is crucial to diagnosis, with radiographs typically revealing epiphyseal irregularities, fragmentation and sclerosis, whereas magnetic resonance imaging enables the early detection of bone marrow oedema, subchondral fractures and cartilage abnormalities. We report on an 11.5-year-old boy presenting with a history lasting for more than 12 months of left ankle pain and bilateral osteochondrosis of the distal tibial epiphysis. The etiology of distal tibial osteochondrosis remains unclear, with proposed mechanisms including vascular disorders, repetitive microtrauma and genetic predisposition. The condition's rarity, particularly in the bilateral form, suggests a multifactorial pathogenesis going beyond mechanical stress alone. Its prognosis depends on the degree of epiphyseal involvement and the presence of premature physeal closure, emphasizing the importance of early diagnosis and tailored management strategies. Based on this rare case, we summarize more than 75 years of observations of patients with osteochondrosis of the distal tibial epiphysis, discuss its etiology and revise information about its known clinical features, radiological characteristics, pathogenesis and treatment.

## 1 Introduction

Osteochondrosis of the distal tibial epiphysis is an exceedingly rare bone disorder, knowledge of which is limited to the very few cases reported in the literature (1–9). Because of the condition's exceptional nature, it is difficult to be affirmative when describing its symptoms. Nevertheless, reported observations indicate that the insidious, progressive onset of ankle pain and swelling generally occurs without any apparent trauma (1–9). Imaging plays a key role in diagnosing the condition. Conventional radiography typically reveals significant irregularities in the distal tibial epiphysis, which appears fragmented and sclerotic, with deformities affecting both planes of the joint. Magnetic resonance imaging (MRI) provides clinicians with the most information since it can detect early changes, such as bone marrow oedema, subchondral fractures and cartilage abnormalities, thus facilitating prompt diagnosis (10).

The aetiology of osteochondrosis of the distal tibial epiphysis remains elusive, and a debate on this topic continues. Proposed pathophysiological mechanisms include an epiphyseal vascular disorder, repetitive microtraumas or mechanical stressors that affect the developing epiphysis and lead to avascular necrosis. However, it is likely that more than one of these mechanisms must occur to explain the vascular disorders that will lead to subsequent osteonecrosis.

The lack of reported cases makes it impossible to establish a standard treatment protocol. Conservative management, including immobilizing the ankle and activity modification, makes sense since it has been reported to result in positive outcomes. However, in certain situations involving persistent pain or severe functional impairment, a surgical procedure may be considered.

We report the case of an 11.5-year-old boy who was referred to us because of persistent pain in the left ankle and radiographical images suggestive of osteochondrosis of the distal tibial epiphysis. To the best of our knowledge, this is only the twelfth reported case of this disorder. We summarize the other 11 cases, discuss their etiology and revise information on their clinical features, pathogenesis and treatment.

## 2 Case report

An otherwise healthy 11.5-year-old boy was referred to our university hospital's pediatric orthopedics department due to a history of pain in his left ankle lasting for more than 12 months. As a soccer player (right foot dominant), the patient reported several previous trivial traumas that did not require any orthopedic treatment. However, a few weeks before the consultation, a new trauma of this ankle occurred, resulting in more sustained symptoms and a function's restriction. The patient underwent an x-ray examination for the first time, and the ankle was immobilized in an orthopedic boot due to a suspected fracture. The patient was referred to an orthopedist who performed a computed tomography (CT) scan and then referred him, in turn, to our pediatric orthopedics outpatient department.

**Figure 1:**
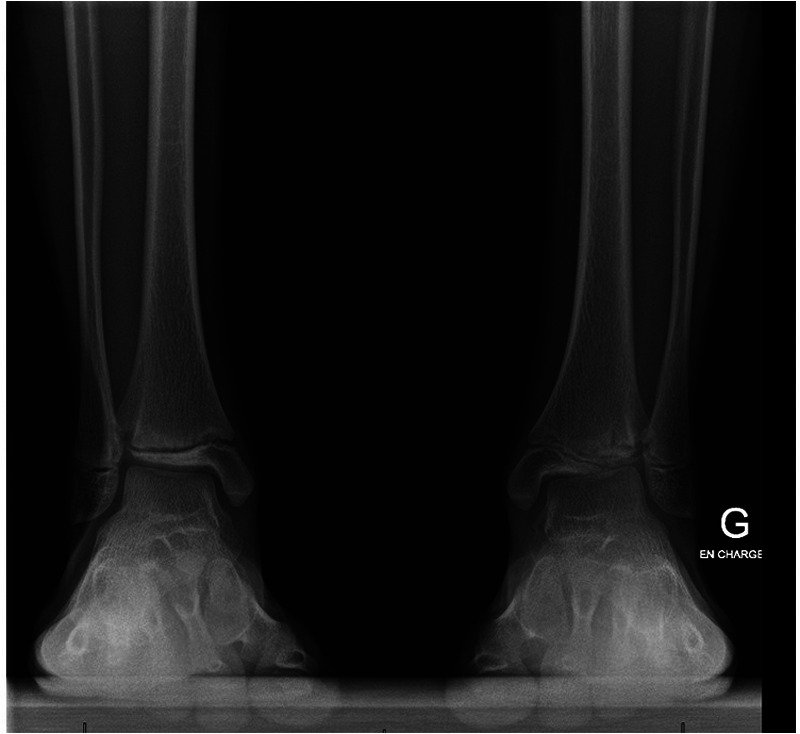
This bilateral standing x-ray demonstrated a valgus of the left distal tibial epiphysis (a distal lateral tibial angle of 76°). Unexpectedly, we also noted trophic changes in the right tibial epiphysis.

**Figure 2:**
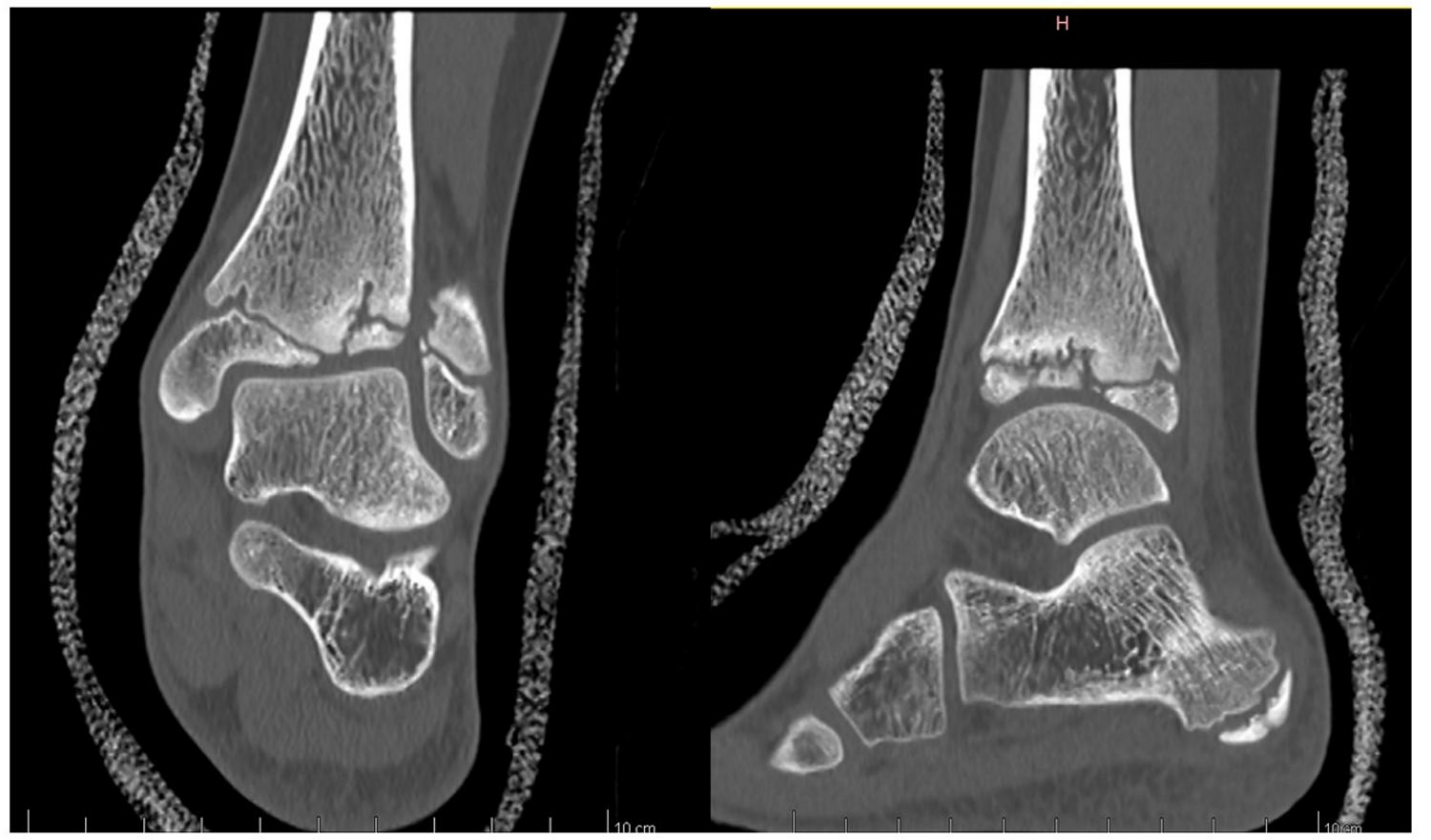
This CT scan of the left ankle more precisely revealed the extent of the damage to both the epiphysis and the physis, demonstrating epiphyseal fragmentation with physeal disruption and the presence of physeal bars.

**Figure 3:**
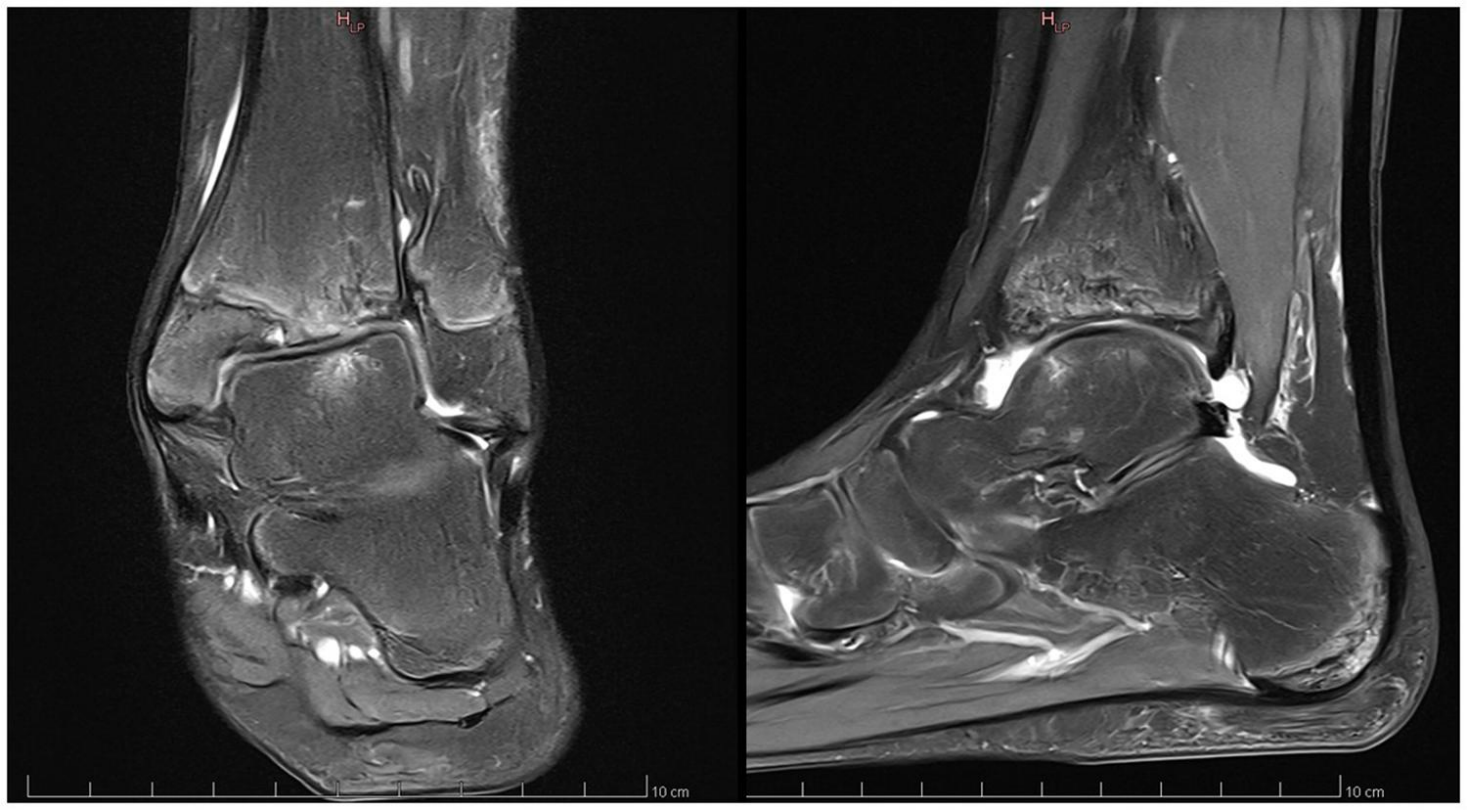
This MRI of the left ankle confirmed the epiphyseal fragmentation, the physeal abnormalities and the metaphyseal deformities.

Anamnesis showed no family history of illness, genetic disorders, or osteochondrosis. The patient was 148 cm tall and weighed 37.5 kg, both measurements being one standard deviation above the mean. Clinical exam showed unrestricted, and symmetrical motion in the left ankle. There was a 13 mm leg length discrepancy on the left, and a standing hindfoot valgus angle of 10°. Palpation triggered pain in the joint's anterolateral aspect. Pain was noted over the anterior talofibular ligament with palpation, tension, weight-bearing, and tiptoe walking. The initial x-ray image of the left ankle revealed an irregularity of the distal tibia, with thinning or even an absence of the lateral epiphysis. The physis itself appeared scalloped and was difficult to clearly distinguish on its lateral side. A standing x-ray image of both ankles was taken, and radiographic alignment analysis revealed valgus of the joint line of the left ankle, with a mechanical distal lateral tibial angle measured at 76° (Figure 1). Unexpectedly, we identified trophic disturbances in the distal epiphysis of the contralateral tibia, indicating the presence of bilateral osteochondrosis. Indeed, the right epiphysis appeared to be both atrophic and sclerotic, but without fragmentation. A CT scan confirmed the fragmentation of the left epiphysis and a complete disruption of the physis on its lateral side. In certain instances, the imaging findings were indicative of possible physeal bars (Figure 2). MRI also confirmed the fragmentation and subsequent involution of the distal tibial epiphysis. It also revealed severe focal alterations in the physis and important metaphyseal deformities consecutive to abnormal endochondral ossification, as well as serious structural alterations in the articular cartilage (Figure 3). No lateral ligamentous or syndesmotic instability, nor rupture, was evident. A diagnosis of bilateral osteochondrosis of the distal tibial epiphysis was made, and the patient was prescribed a symptomatic treatment involving a prohibition of any sports activities and temporary immobilization in a left plaster cast for four weeks without weightbearing due to pain.

**Figure 4:**
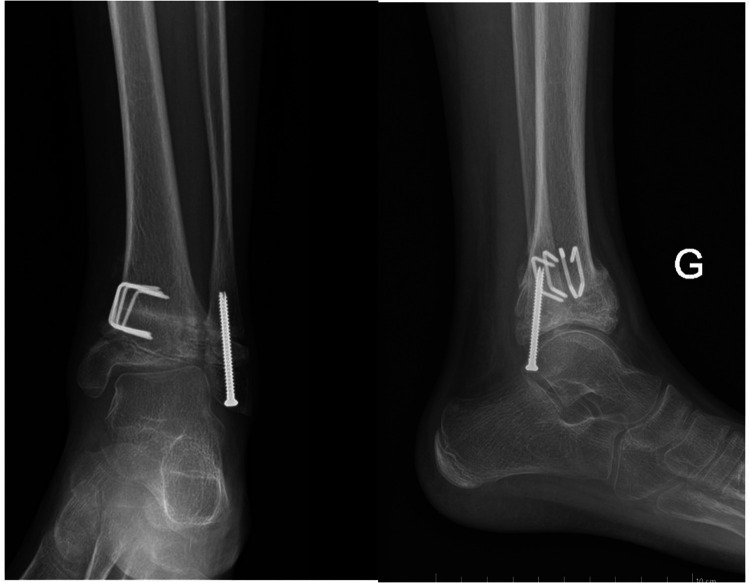
This final postoperative x-ray shows the left ankle after an arthroscopy that had revealed irregular joint cartilage and the subsequent osteotomy of the left tibial metaphysis to correct the varus with an additional epiphysiodesis of the distal tibia and a complete epiphysiodesis of the distal fibula.

Despite this, however, the patient noted no relief from pain. Even though joint function was preserved, marked functional pain persisted. As a result, surgical treatment was suggested to the boy's parents, and they gave their consent. The treatment started with a diagnostic arthroscopy of the left ankle; during the arthroscopy, we noted irregularities and fissuring in the articular cartilage of the distal tibia, but there were no mobile fragments or intra-articular loose bodies. Then a 13° distal tibial medial varisation osteotomy with bone subtraction (7 mm) was realized to correct the valgus, and a complementary epiphysiodesis was performed at the level of the distal tibia by drilling carefully the medial aspect of growth plate under radiographic guidance. A distal fibular epiphysiodesis was also performed by multiple direct drilling of the physis and by inserting a fully threaded screw through the growth plate (Figure 4).

The ankle was then immobilized in a short leg fiberglass cast for 6 weeks with 3 weeks of strict unloading followed by progressive weightbearing during the following 3 weeks. After the removal of the cast, the patient underwent physiotherapy sessions for 6 weeks. At the 4th postoperative month, the patient began to actively mobilize his ankle in the swimming pool and on an exercise bike. Finally, at the 6th postoperative month, he has resumed football training and will resume matches in September (9 months postoperative).

Ankle-centered radiographs are currently obtained every three months, while EOS imaging in the standing position is scheduled every six months. Accordingly, limb length discrepancy will be closely monitored over the upcoming months and years, and, if indicated, growth modulation will be implemented, if necessary, on the contralateral limb at the appropriate time.

## 3 Discussion

Osteochondrosis can be a confusing term as it covers a group of self-limiting bone development anomalies that primarily involve the ossification centres of the epiphysis and the apophysis. The term *osteochondritis* is also frequently used in the literature as a synonym of osteochondrosis (9); however, it appears somewhat of a misnomer since histological studies have failed to support an inflammatory aetiology (11). Osteochondrosis is probably a less misleading and more appropriate term than osteochondritis (12).

Although its precise pathophysiology remains elusive, the main factors responsible for osteochondrosis have been identified and revolve around primary vascular disorders, acute traumatic events or multiple repetitive traumas. Alongside these, certain hereditary, neurological and nutritional mechanisms have been proposed to explain the pathogenesis of epiphyseal osteochondrosis (13, 14). In addition, some metabolic and histological alterations, such as abnormal mineralisation, altered collagen II content, abnormal extracellular matrix proteins and alterations in collagen crosslinking, have been reported and are considered to be determinant pathogenic factors (15, 16). Several scenarios can thus be evoked: osteochondrosis could be observed in a normal epiphysis that has been subjected to extreme trauma; it could occur in a mildly dyschondrotic epiphysis exposed to greater-than-usual stress; or it could occur in a severely affected dyschondrotic epiphysis subjected to physiological stress (17).

Whatever the mechanism, the osteochondral process results in ischemic necrosis of the ossification centre, leading to a degeneration of the epiphyseal or apophyseal osseous nucleus. Osteochondrosis occurs after the bony nucleus appears in the epiphysis, and since it is mainly cartilaginous—because it grows rapidly and can be subjected to significant mechanical pressures—its cells are more vulnerable and prone to necrosis. This results in a local failure of endochondral ossification, either within the articular epiphyseal growth cartilage or the articular apophyseal growth cartilage.

Osteochondrosis generally begins in childhood as a necrotic and degenerative condition. The osteochondral process can affect the entire epiphysis or apophysis and involve either a single epiphysis or several. The disease can also be sequential or simultaneous. Osteochondrosis is more frequent in males, and this phenomenon may be attributed to the facts that ossification centers naturally appear later in males and that boys are more exposed to physical traumas and stresses in early childhood (17).

Paradoxically, osteochondrosis of the distal tibial epiphysis is one of the most rarely affected epiphyses in the body. This case is only the twelfth reported osteochondrosis of the distal tibial epiphysis and only the fourth to appear in its bilateral form (3, 8, 9). Less severe forms of osteochondrosis have also been described, solely involving the medial malleolus (18–20). The ankle joints bear the full body weight during daily activities, subjecting them to significant articular forces and increasing the risk of epiphyseal overload from a mechanical perspective. We might thus expect the distal tibial epiphysis to be far more frequently affected if mechanical stresses (such as trauma, repetitive microtrauma or excessive overload) played a key precipitating factor in the pathogenesis of osteochondrosis. The low prevalence of osteochondrosis of the distal tibia and the occurrence of bilateral lesions suggest a hypothesis that goes beyond trauma alone, and this should make us think about whether genetic factors are involved in the condition's pathogenesis. We conclude that vascular, traumatic, and hereditary factors must combine to cause osteochondrosis of the distal tibial epiphysis; trauma alone is likely insufficient. In this specific context, Turati et al.'s hypothesis makes sense: anomalous hereditary features and a probable congenital alteration of the blood supply should be considered as probable cofactors explaining a bilateral osteochondrosis of this specific anatomical region (9).

The appearance of the epiphysis on imaging is closely correlated with the severity and stage of the disorder. Conventional radiography is essential to any initial assessment as it can reveal sclerosis, fragmentation or even a collapse of the distal tibial epiphysis. Unlike certain sites where fragmentation of the epiphysis or apophysis can be seen in healthy asymptomatic individuals, fragmentation of the distal tibial epiphysis must be considered pathological. Osteonecrosis initially manifests via a low signal intensity in T1-weighted imaging and via higher signal intensities in T2-weighted and short tau inversion recovery (STIR) imaging (10). As osteochondrosis progresses, it is typically seen as a low-intensity signal in T1- and T2-weighted imaging because of the advancing sclerosis, usually accompanied by subchondral flattening, collapse and fragmentation. MRI is especially crucial for evaluating any disruption of the physis, the failure of endochondral ossification and growth disorders, all of which frequently lead to premature physeal closure (10).

Since osteochondrosis is currently considered a self-limiting disease related to a benign condition, osteochondrosis of the distal tibial epiphysis is primarily managed by using rest to avoid weight-bearing while maintaining a good range of motion of the ankle joint (4). Some authors have suggested drilling the area of epiphyseal sclerosis to stimulate revascularisation, but they have not presented any scientific evidence of this intervention's efficacy (4, 19). In cases like the present one, there may be a severe alteration in the shape of the affected lesion due to both asymmetrical necrosis of the epiphysis and premature physeal closure. Thus, every effort should be made to obtain a congruent, mobile, painless joint. In this regard, realignment osteotomy of the distal tibia may be suggested to a young patient's family to improve the ankle's static equilibrium, balance the mechanical constraints within it and reduce secondary deformations (4, 19).

Prognosis is correlated to the severity of the disease, to the amount of epiphyseal involvement and, above all, to the alteration of the joint's shape. The greater the potential for growth of the affected epiphysis and the younger the patient's age at the onset of osteochondrosis, the more likely a reconstruction with remodelling of the damaged epiphysis will have a favourable outcome.

## 4 Conclusion

Osteochondrosis of the distal tibial epiphysis is a rare and poorly understood condition that can lead to progressively growing pain, functional impairment and joint deformity. The pathogenesis likely involves a combination of vascular disruption, repetitive microtrauma and hereditary factors rather than mechanical stress alone. Bilateral cases, as observed in our patient, further support the hypothesis of an underlying genetic or congenital predisposition. Diagnosis relies heavily on imaging, with radiographs, computed tomography and magnetic resonance imaging playing complementary roles in assessing the integrity of the epiphysis, physeal structure and cartilage health. While conservative management remains the first-line approach, persistent pain or significant epiphyseal deformation may necessitate a surgical intervention—such as an arthroscopy to remove loose bodies or facilitate regeneration by applying a microfracture technique, epiphysiodesis to avoid progressive joint misalignment and redirectional osteotomy to improve joint alignment—that can help restore joint function and prevent secondary degenerative changes. Given the exceptional rarity of this disease, further case reports and long-term studies are needed to better understand its natural history, optimise treatment strategies and refine prognosis assessments.

## Data Availability

The original contributions presented in the study are included in the article/Supplementary Material, further inquiries can be directed to the corresponding author.
